# Nurse Retention in Hospitals: A Multilevel Integrative Review of Organizational Determinants

**DOI:** 10.3390/healthcare14060772

**Published:** 2026-03-19

**Authors:** Assunta Guillari, Marco Abagnale, Chiara Palazzo, Maria Assunta Fulco, Teresa Rea, Vincenza Giordano

**Affiliations:** 1 Department of Translational Medical Sciences, Clinical Research Center DEMeTra, University of Naples “Federico II”, 80131 Naples, Italy; assunta.guillari@unina.it; 2 Department of Critical Care, Unit of Anesthesiology and Intensive Care, M. Scarlato Hospital, 84091 Salerno, Italy; abagnale.marco@gmail.com; 3 Department of Oncology, Hematology and Cellular Therapies, Santobono Pausilipon Hospital, 80123 Naples, Italy; 4 Nursing and Midwifery Sciences Program, University of Naples “Federico II”, 80131 Naples, Italy; assunta_92@hotmail.it; 5 Department of Public Health, University of Naples “Federico II”, 80131 Naples, Italy; teresa.rea@unina.it (T.R.); enza-giordano@hotmail.it (V.G.)

**Keywords:** nurse retention, organizational factors, work environment, transformational leadership, workforce sustainability

## Abstract

**Highlights:**

**What are the main findings?**

- Nurse retention is a multidimensional phenomenon driven by the interaction of organizational conditions, relational dynamics, and individual psychological resources.
- Transformational and participative leadership consistently emerge as key determinants of nurses’ intention to stay across hospital settings.

**What are the implications of the main findings?**

- Retention strategies focused solely on structural improvements are insufficient without parallel investment in relational climates and individual well-being.
- Integrated, theory-informed approaches are essential to support workforce sustainability and long-term quality of care.

**Abstract:**

**Background/Objectives**: Nurse retention remains a major global challenge for healthcare systems, intensified by workforce aging, rising care complexity, and the long-term impact of the COVID-19 pandemic. Despite extensive research, the evidence on nurse retention remains fragmented and frequently focuses on isolated determinants. This review aimed to synthesize the multifactorial determinants of nurse retention by integrating organizational, relational, and individual perspectives. **Methods**: An integrative review was conducted following Whittemore and Knafl’s approach and reported according to PRISMA 2020 guidelines where applicable. A systematic search of six databases identified studies published between 2016 and 2026 addressing nurse retention in hospital settings. Included studies underwent methodological quality appraisal using validated tools, and findings were synthesized narratively. **Results**: Twenty-five articles were included. The analysis revealed differences in perspective between nurse managers and nurses regarding the factors that influence retention. Transformational and participative leadership among nurse managers enhanced staff retention through supportive organizational climates and higher professional commitment. For staff nurses, positive work environments, collegial support, and psychological resources such as self-efficacy and resilience were key predictors of intention to stay. These findings can be interpreted through Herzberg’s Two-Factor Theory, Self-Determination Theory and Theory of Planned Behavior, which collectively highlight how recognition, autonomy, and competence satisfaction drive nurses’ intention to remain in their roles. **Conclusions**: Nurse retention reflects dynamic, multilevel processes rather than the influence of single determinants. Integrated, theory-informed approaches targeting organizational structures, relational climates, and individual psychological resources are required to strengthen workforce sustainability and support high-quality care delivery.

## 1. Introduction

The shortage of nursing staff represents one of the most significant challenges for healthcare systems worldwide. According to estimates by the World Health Organization [1], the global nursing deficit decreased from 6.2 million in 2020 to 5.8 million in 2023, with a further reduction to 4.1 million projected by 2030 [1]. Despite this trend, the literature highlights how this shortage remains a structural issue, mainly associated with the aging of the workforce, the reduced attractiveness of the nursing profession, with a consequent decrease in enrollments in training programs, the increase in burnout among healthcare professionals following the COVID-19 pandemic, and the growing demand for healthcare linked to the prevalence of chronic diseases [2]. Population aging and the increase in chronic diseases contribute to a higher demand for care, requiring an ever-growing number of nurses who are adequately trained to respond to emerging care needs [3]. These structural factors have been further amplified by the COVID-19 pandemic, which has exacerbated pre-existing challenges and highlighted the urgent need to improve the working conditions of nursing staff in order to ensure sustainable and effective professional practice [4]. Within this context, the capacity of healthcare organizations to retain qualified nursing staff has emerged as a key determinant of the sustainability and effectiveness of health systems globally. The World Health Organization urges governments to take action during the 2026–2030 period, including the expansion and equitable distribution of nursing jobs, the strengthening of education and training systems, the improvement of working conditions and well-being, the development of regulation and advanced roles, the promotion of gender equity, preparedness for climate and digital challenges, and support for nursing leadership [1].

Nurse retention refers to an organization’s ability to maintain nursing staff within the workforce overtime [5]. The stability of the nursing workforce is an essential condition for ensuring the quality and sustainability of care, given the central role nurses play in achieving health system objectives. In particular, nurses ensure continuity of care for hospitalized patients by responding in an integrated manner to both their physical and emotional needs [6]. A possible effect of professional distress among nurses is voluntary turnover, which contributes to worsening staff shortages: high levels of turnover undermine the stability of care teams, hinder the transfer of skills, and increase the risk of clinical errors, with negative repercussions both on health outcomes and on the patient experience [6,7]. Numerous studies suggest that nurse retention is influenced by multiple factors shaping nurses’ intentions to remain in their work setting, including characteristics of the work environment, leadership models, levels of burnout, and job satisfaction [8,9,10].

The determinants of nurse retention can be more thoroughly understood through the contribution of theoretical models used in organizational psychology. In particular, Herzberg’s Two-Factor Theory distinguishes between hygiene factors, such as pay, working conditions, and safety, and motivational factors, including recognition, empowerment, and opportunities for professional growth, providing an interpretative framework for analyzing the role of organizational conditions in the work context [11]. Self-Determination Theory (SDT) emphasizes three fundamental psychological needs—autonomy, competence, and relatedness—considered essential for the optimal functioning of individuals in organizational contexts. This theory offers a useful perspective for understanding how the relational characteristics of the work environment can influence motivation and professional behavior [12,13]. Finally, the Theory of Planned Behavior (TPB) offers a theoretical framework for analyzing nurses’ intention to remain through the interaction of individual attitudes, subjective norms, and perceived behavioral control, providing an interpretative model that integrates both personal and contextual dimensions [14]. Despite the extensive scientific production on the phenomenon of nurse retention, the literature is fragmented and predominantly focused on individual determinants, which are analyzed separately at the organizational, relational, or individual level [5,7,8]. Existing reviews rarely provide an integrated synthesis that incorporates relevant theoretical models. Consequently, a knowledge gap persists in the comprehensive understanding of this phenomenon, limiting the development of evidence-based retention strategies. Considering these limitations, an integrative review is warranted to synthesize the determinants of nurse retention by jointly examining organizational, relational, and individual factors.

## 2. Materials and Methods

### 2.1. Study Design

This integrative review was conducted according to the methodological approach outlined by Whittemore and Knafl (2005) [15], which allows for the inclusion, analysis, and critical synthesis of heterogeneous evidence in order to build a holistic and in-depth understanding of the phenomenon under investigation. This methodological approach is characterized by its capacity to integrate studies with different designs, enabling a comprehensive and multidimensional understanding of the phenomenon under investigation. The aim of this study is to synthesize the multifactorial determinants of nurse retention by examining organizational, relational, and individual perspectives, thereby informing the development of evidence-based retention strategies to support workforce sustainability. The study adhered, where applicable, to the Preferred Reporting Items for Systematic Reviews and Meta-Analyses (PRISMA 2020) guidelines [16] and followed six iterative phases: (1) formulation of a focused research question; (2) development of a systematic search strategy; (3) application of inclusion and exclusion criteria; (4) critical appraisal of the methodological quality of the included studies; (5) data extraction, thematic categorization, and narrative synthesis; (6) integrative interpretation of the findings across the different sources.

### 2.2. Research Strategy

Literature searches were conducted in six databases: PubMed, CINAHL Complete, Scopus, Cochrane Library, APA PsycInfo, and APA PsycArticles, including studies published between January 2016 and January 2026, with a focus on the multifactorial determinants of nurse retention. The research question was formulated using the PEO model (Population, Exposure, Outcome) proposed by Bettany-Saltikov (2012) [17], as follows:

- Population (P): nurses
- Exposure (E): organizational, relational, and individual factors
- Outcome (O): nurse retention

The main search terms used were: “personnel retention,” “staff retention,” “nurse retention,” “working conditions,” “workplace,” “work environment,” and “healthcare.” The use of the Boolean operators AND and OR made it possible to combine MeSH terms and free-text keywords, facilitating the identification of the most relevant studies. The search strings (Table S1) were tested and subsequently optimized to ensure the best possible balance between sensitivity and specificity, reducing the risk of including irrelevant results.

### 2.3. Inclusion and Exclusion Criteria

Inclusion criteria:

1. Primary studies and secondary research (systematic reviews, scoping reviews, or integrative reviews) addressing the topic of nurse retention.
2. Studies published in English.
3. Research conducted in the field of hospital nursing, including both public and private healthcare facilities.
4. Studies that analyze the multifactorial determinants of nurse retention, including organizational, relational, and individual dimensions.
5. Articles that explicitly address nurse staff retention as a primary or secondary outcome.

Exclusion criteria: Studies presenting one or more of the following characteristics were excluded from the review:

1. Studies that included mixed populations without a specific analysis for nurses.
2. Editorials, commentaries, opinion articles, and conference abstracts
3. Articles not available in full-text format or published outside the defined time frame (January 2016–January 2026).

### 2.4. Selection of Studies

The study selection process initially led to the identification of 1327 articles from the consulted databases (CINAHL: n = 398; PubMed: n = 482; APA PsycArticles/APA PsycInfo: n = 12; Scopus: n = 340; Cochrane Library: n = 85). All records were imported and managed using the Rayyan—Intelligent Systematic Review software (https://www.rayyan.ai, accessed on 10 January 2026), to optimize the screening phase and reduce the risk of selection bias. After importing the records, duplicates were removed, allowing 1118 articles to be assessed by reading titles and abstracts, carried out independently by two reviewers (M.A., C.P.). The Rayyan software was used to classify the studies into two categories, included and excluded, while maintaining the blind review mode between the reviewers, which was deactivated at the end of the screening phase. Any discrepancies that arose during the selection process were resolved through discussion between the reviewers and, when necessary, with the involvement of a third reviewer (A.G.).

**Figure 1:**
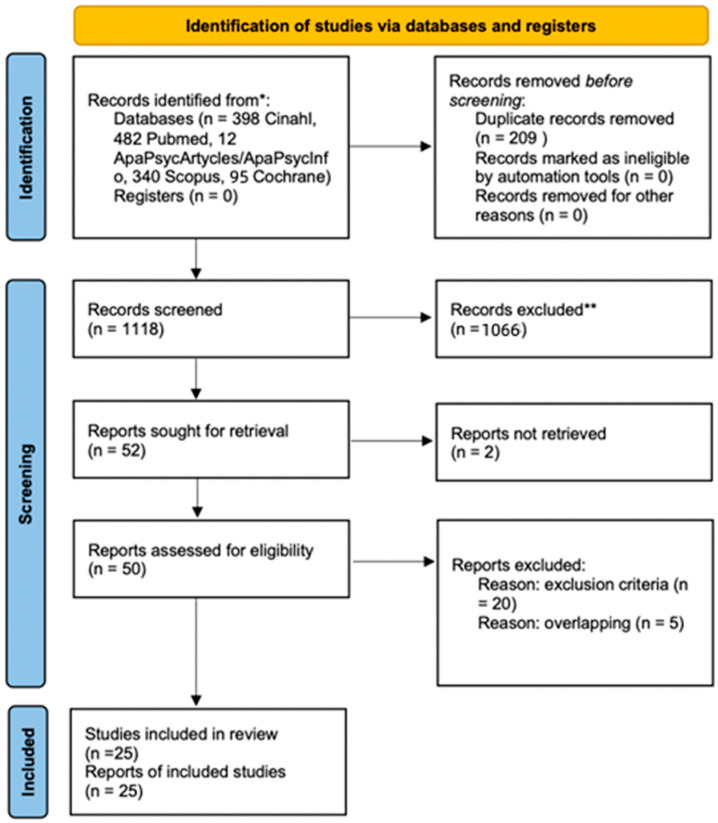
PRISMA flow chart. * Consider, if feasible to do so, reporting the number of records identified from each database or register searched (rather than the total number across all databases/registers). ** If automation tools were used, indicate how many records were excluded by a human and how many were excluded by automation tools.

Following the preliminary screening, 50 articles were selected for full-text assessment, as they were considered potentially eligible according to the predefined inclusion and exclusion criteria. Subsequent full-text review led to the exclusion of 25 studies: 20 did not meet the inclusion criteria, and 5 primary studies were already included in the selected reviews and were therefore excluded to avoid data duplication. The entire process of study identification, screening, eligibility assessment, and final inclusion was documented and graphically presented using the PRISMA 2020 flow diagram (Figure 1), adapted to the methodological context of the integrative review in accordance with PRISMA guidelines [16].

### 2.5. Quality Appraisal

The assessment of methodological quality and risk of bias was conducted in accordance with the methodological principles of integrative reviews, which allow for the synthesis of heterogeneous evidence while maintaining appropriate standards of rigor and transparency [15]. Due to the heterogeneity of the included study designs, validated critical appraisal tools specific to each study design were employed to assess methodological quality. Qualitative studies and literature reviews were evaluated using the Quality Appraisal for Diverse Studies (QuADS) tool [18], which provides a maximum score of 39 points and predefined thresholds for classifying methodological quality (high: 30–39; moderate: 20–29; low: <20). Quantitative cross-sectional studies were instead assessed using the JBI Checklist for Analytical Cross Sectional Studies (JBI, 2025) [19], with scores ranging from 0 to 8 and quality classified as high (7–8), moderate (5–6), or low (<5). The evaluation of the studies was carried out independently by two reviewers; any discrepancies were resolved by consensus, ensuring inter-reviewer agreement. The entire methodological appraisal process is summarized in Table S2a,b.

### 2.6. Extraction of Results and Synthesis of Studies

A total of 25 studies were included, comprising 14 quantitative studies, 6 qualitative studies, and 5 literature reviews. To ensure a clear, systematic, and consistent synthesis of the included studies, data were extracted and organized into a thematic table (Table S3), which reports the following elements: author(s), year of publication, study design, objective/focus, sample and setting characteristics, main findings, reported limitations, and methodological quality. This structured tabular format was chosen to facilitate both intra-method comparison and inter-method integration across different methodologies [20], thereby ensuring a transparent synthesis and improving the interpretability of the extracted data [21].

## 3. Results

### 3.1. Characteristics of the Studies

Overall, 25 studies were included in the review, of which 14 were quantitative [22,23,24,25,26,27,28,29,30,31,32,33,34,35], 6 qualitative [36,37,38,39,40,41], and 5 literature reviews [42,43,44,45,46] (Table S3). The methodological distribution reflects the multidimensional nature of the nurse retention phenomenon, with a predominance of cross-sectional descriptive and correlational designs. The studies were conducted across diverse geographical contexts, including both high- and middle-income countries such as the United States, Canada, the United Kingdom, China, Saudi Arabia, Ghana, India, and Thailand, indicating good international representativeness. The hospital setting was predominant, consistent with the inclusion criteria, encompassing both public and private institutions as well as university and non-university hospitals. Some studies specifically focused on specialized contexts such as emergency departments, intensive care units, and perioperative settings [29,32,36,39,44]. Overall, the methodological quality of the included studies was generally high: among the quantitative studies assessed with the JBI Checklist, 9 were classified as high quality and 5 as moderate quality. The qualitative studies and reviews were generally judged to be of high methodological quality according to the QuADS tool, with scores ranging from 32 to 39. Regarding the study population, the included studies primarily involved nursing staff, with a non-negligible number of investigations focusing on leadership and managerial roles, particularly those of nurse managers [22,30,35,43].

This distinction guided the subsequent categorization of the findings, structured into two macro-areas:

- Studies focused on nursing managers (nurse managers and leaders);
- Studies focused on nursing staff (staff nurses).

Within each macro-area, the results were further organized according to three analytical dimensions consistent with the objectives of the review:

- Organizational factors, relating to structure, work environment, and management practices;
- Relational factors, concerning interpersonal and leadership dynamics;
- Individual factors, linked to the psychological and motivational characteristics of nurses.

Narrative synthesis was used to integrate the findings, while conceptual triangulation was employed to validate them by cross-checking evidence from different methodological sources. To facilitate the interpretation of the results, a summary of the main determinants of nurse retention identified across the included studies is presented in Table 1. The determinants are organized according to the perspectives of nurse managers and staff nurses and across the organizational, relational, and individual dimensions.

### 3.2. Studies Focused on Nursing Managers (Nurse Managers and Leaders)

Studies that have examined nurse retention from the perspective of nurse managers primarily focus on the influence of leadership styles, organizational climate, and managerial behaviors on staff retention. Overall, the evidence converges in recognizing leadership quality as a crucial determinant of retention, operating through closely interconnected organizational, relational, and individual dimensions [22,30,35,43].

#### 3.2.1. Organizational Factors

At the organizational level, transformational and participative leadership emerges as one of the main determinants of staff retention. The study by Adalin et al. (2025) [22] showed that the transformational competence of nurse managers is significantly associated with nurses’ intention to stay (r = 0.22, *p* < 0.001). All dimensions of transformational leadership—idealized influence, inspirational motivation, intellectual stimulation, and individualized consideration—together with specific transformational behaviors, such as articulating a compelling vision, providing individualized support, promoting intellectual stimulation, and modeling ethical practice, are positively associated with nurse retention, confirming the role of leadership as a central motivational mechanism. Consistently, the study by Ofei and Paarima (2022) [35], conducted in 38 hospitals in Ghana, found that transformational and participative leadership styles of nurse managers were positively associated with nurses’ intention to stay, whereas transactional, autocratic, and laissez-faire styles were negative predictors of intention to remain. Transformational leadership explained a significant proportion of the variance in intention to stay (20.9%), suggesting that managers’ ability to motivate, inspire, and foster professional engagement represents a strategic lever for staff retention. From a synthesis perspective, the integrative review by Goens and Giannotti (2024) [43] further strengthened this evidence, showing that transformational leadership does not act primarily directly on retention, but rather operates through the improvement of the work environment, job satisfaction, and organizational commitment, which mediate its effect on intention to stay.

#### 3.2.2. Relational Factors

On a relational level, the quality of interactions between nurse managers and nursing staff emerges as a crucial determinant of nurses’ continued employment.

The study by Hossny et al. (2023) [30], conducted in two Egyptian hospital facilities, showed that a favorable organizational climate and relationships characterized by mutual support are significant predictors of higher levels of intention to remain in the workplace. Conversely, the presence of toxic leadership behaviors—such as authoritarianism, unpredictability, and marked self-promotion—is associated with a significant reduction in retention rates. The authors also emphasize that clarity of expectations and consistent leader behavior are necessary conditions for fostering organizational trust and a sense of belonging to the work environment.

#### 3.2.3. Individual Factors

The included studies also indicate that retention is influenced by intrapersonal characteristics and by specific motivational competencies of nurse managers. Adalin et al. (2025) [22] highlights how a leader’s individual competence in communicating a shared vision and systematically recognizing staff members’ needs fosters the development of a climate of trust and self-fulfilment, which is associated with a greater intention to remain within the organization. Similarly, Ofei and Paarima (2022) [35] confirm that participative leadership, characterized by practices of active listening and a high level of staff involvement in decision-making processes, strengthens nurses’ sense of autonomy and professional responsibility. The review by Goens and Giannotti (2024) [43] integrates and consolidates this evidence, emphasizing that leaders’ individual qualities represent crucial resources for staff retention at both the individual and organizational levels. Overall, studies focusing on the role of the nurse manager consistently indicate that nurse retention is closely associated with leadership quality and the relational climate fostered by managers. The available evidence suggests that transformational and participative leadership styles contribute significantly to the creation of sustainable work environments, promoting organizational well-being, a sense of belonging, and opportunities for professional development and growth [22,30,35,43].

### 3.3. Studies Focused on Nursing Staff (Staff Nurses)

Studies examining nurse retention from the perspective of nursing staff describe a complex and multifactorial phenomenon shaped by the interaction between organizational conditions, professional relational dynamics, and individual characteristics. The available evidence consistently indicates that the quality of the work environment, managerial support, career development opportunities, and psychological well-being are key determinants of nurses’ intention to remain in their positions.

#### 3.3.1. Organizational Factors

Organizational factors constitute the most extensively explored dimension. Numerous quantitative studies [23,26,28] have shown that the work environment and the structural characteristics of the organization exert a direct influence on staff retention processes. Settings perceived as favorable, with adequate staffing levels, a high degree of professional autonomy, opportunities for development, and formal recognition, are associated with higher job satisfaction and a greater intent to stay. In the contribution by Blegen et al. (2017) [26], which used the Practice Environment Scale of the Nursing Work Index (PES-NWI), the McCain Intent to Stay Scale, and the Global Job Satisfaction Survey by Quinn and Shepard, a statistically significant association was found between the work environment and the intention to remain in service (t = 4.83, *p* < 0.001). In that study, the organizational context emerged as an independent predictor of both job satisfaction and retention, while the type of hospital, more than the type of ward, emerged as the variable associated with the willingness to remain. Eltaybani et al. (2018) [28], in a survey conducted on a sample of more than 3000 nurses working in Japanese long-term care units, showed that managerial support, training opportunities, and perceived quality of care are significant predictors of the intention to remain in service, highlighting the crucial role of an organizational culture orientated toward learning and continuous improvement. Systematic and integrative reviews [42,44,45] have further corroborated the multilevel nature of organizational determinants, identifying supportive leadership, policies aimed at promoting organizational well-being, and workplace safety conditions as the main cross-cutting determinants of nurse retention. Farahani et al. (2024) [42] documented that, in the context of the COVID-19 pandemic, the availability of personal protective equipment, access to structured psychological support interventions, and the perception of workplace safety were key factors in containing intentions to leave the profession. Consistently, McIntyre et al. (2024) [44] highlighted, in emergency and urgent care settings, the role of organizational support structures and clinical transition programs in mitigating turnover. Finally, Phakdeechanuan et al. (2025) [45] identified institutional support, organizational flexibility, and systematic opportunities for professional development as consistent and robust predictors of nurse retention.

#### 3.3.2. Relational Factors

Relational factors represent a crucial dimension in the processes that influence the retention of nursing staff and include the quality of interpersonal relationships, the degree of team cohesion, mutual trust, and the level of perceived support from colleagues and supervisors. Available evidence indicates that a positive relational climate, characterized by open communication, mutual respect, and professional recognition, significantly contributes to increased job satisfaction and strengthens nurses’ intention to remain in their positions [33,34,36,37,38,40,44,45,46]. The quantitative contribution by Kao and Kao (2024) [33], grounded in the Job Demands–Resources model, highlighted how relational resources—in particular peer support and trust placed in proximal leadership—promote increased engagement and job satisfaction, which, in turn, positively mediate the intention to remain within the organization. Similarly, Mulkey and Casey (2023) [34], analyzing the onboarding processes of recent graduates in the workplace, found that participants regarded perceived support, mentoring, and recognition from supervisors as a central factor in decision-making processes concerning the choice to remain in service. Qualitative evidence makes it possible to explore in greater depth the relational dynamics described by nurses. Arakelian et al. (2019) [36] found that the sense of belonging to the group and mutual peer support are factors that facilitate resilience, particularly in critical care and perioperative settings. Spoolder et al. (2024) [40] outlined work environments characterized by low-hierarchy relational structures and high cohesion as contexts that are conducive to organizational well-being and job stability. Loft et al. (2020) [38] and Ejebu et al. (2025) [37] also highlighted how the visibility, accessibility and availability of frontline leaders contribute to building trust and to reducing the intention to leave the profession or one’s job. The systematic reviews included corroborate and systematize this empirical evidence [44,45,46]. Pressley and Garside (2023) [46] identify teamwork and effective communication with colleagues and supervisors among the most robust predictors of retention, often showing a greater impact than economic or contractual factors. McIntyre et al. (2024) [44], with specific reference to emergency and urgent care settings, highlight the central role of trust and interprofessional collaboration as key elements for staff stability. Phakdeechanuan et al. (2025) [45] integrate these findings, emphasizing how supportive relationships and a collaborative climate act across the board as determinants of the retention of nursing staff. Overall, the body of evidence emerging from the various lines of research indicates that the quality of interpersonal and professional relationships, both among peers and with frontline leaders, is a crucial determinant in the retention processes of nursing staff.

#### 3.3.3. Individual Factors

Studies exploring personal factors in nurse retention highlight how individual, psychological, motivational, and value-related dimensions play a decisive role in nurses’ intentions to remain. In particular, self-efficacy, resilience, job satisfaction, and emotional well-being emerge as key resources that enable nurses to cope with organizational demands and sustain their professional commitment [24,25,27,31,37,38,39,41,45,46]. Quantitative studies [24,27,31] consistently confirm the protective role of individual psychological resources in work-retention processes. Alshaibani et al. (2024) [24] highlighted that self-efficacy and job satisfaction are positively correlated both with organizational commitment and with the intention to stay, suggesting that the perception of personal competence helps strengthen motivation and occupational stability. In line with these findings, Chua et al. (2025) [27] identified psychological capital—comprising self-efficacy, hope, optimism, and resilience—as one of the strongest predictors of nurse retention. Similarly, Kim and Yoo (2018) [31] reported comparable evidence in a sample of recent graduates, showing that higher levels of psychological capital are associated with greater job satisfaction and stronger commitment to remaining in the profession. Further contributions have examined the role of personal values and the meaning attributed to work. Ashwini and Padhy (2024) [25] demonstrated that workplace spirituality and a sense of professional meaning mediate the relationship between job satisfaction and intention to stay, emerging as important psychological mechanisms supporting the maintenance of the organizational relationship.

Qualitative evidence [37,39,41] outlines a more experiential picture of nurses’ individual lived experience. Ejebu et al. (2025) [37] documented how psychological well-being and the possibility of reconciling work and private life are central determinants in the decision to remain in the profession, both for nurses in the early stages of their careers and for those with greater seniority. In a post-pandemic context, Kilcommons et al. (2025) [39] found that support for mental health and the perception of professional recognition help to strengthen the sense of self-efficacy and to reduce the intention to leave. Washeya and Fürst (2021) [41] showed that intrinsic motivation, stemming from the perceived sense of usefulness and belonging to the professional community, functions as a protective factor against burnout and promotes remaining in the profession even in contexts characterized by limited resources. Integrative and systematic reviews [45,46] confirm that personal resources interact with organizational and relational factors, emerging as an essential level of analysis for understanding the mechanisms of retention.

## 4. Discussion

This integrative review highlights how nurse retention constitutes a complex, multidimensional phenomenon, shaped by the interaction of organizational, relational, and individual factors. The findings indicate that no single dimension, when considered in isolation, can fully explain nurses’ decisions to remain in the profession or within a specific healthcare organization. Rather, retention emerges as the result of a dynamic balance between structural conditions, the quality of professional relationships, and individual resources. A key contribution of this review lies in distinguishing between the perspectives of nursing managers and nursing staff. The results suggest that nurse managers influence retention mainly through the creation of favorable organizational and relational contexts [22,30,35,43], whereas nurses intention to stay on the job as a process that is not only organizational but also subjective, mediated by everyday interpersonal relationships [33,34,36,37,38,40,44,45,46] and by their own psychological resources [24,25,27,31,37,38,39,41,45,46]. This distinction makes it possible to highlight how retention is the outcome of processes that operate simultaneously at different levels of the organizational system. Overall, organizational factors represent the most frequently analyzed dimension and play a structurally decisive role. Favorable work environments, characterized by adequate staffing, high safety standards, opportunities for professional development, and supportive policies, are consistently associated with higher job satisfaction and a greater intention to stay [23,26,28]. This is consistent with Herzberg’s Two-Factor Theory, according to which job satisfaction and dissatisfaction stem from two distinct sets of determinants: hygiene factors, related to the work context, and motivational factors, related to the content of the work and personal fulfillment [11]. Adequate staffing, safety, favorable working conditions, supportive policies, and governance arrangements can be traced back to hygiene factors. In contrast, elements such as professional recognition and opportunities for career development reflect motivational factors and appear crucial in sustaining nurse retention over the long term. The findings suggest that, in the absence of such motivational factors, even structurally adequate organizational environments may not be sufficient to counteract intentions to leave the profession or the organization. Self-Determination Theory (SDT) provides a particularly suitable theoretical framework for interpreting the relational and individual factors emerging from the results. According to this model, self-determined motivation is supported by the satisfaction of three basic psychological needs: autonomy, competence, and relatedness [12,13]. In the present review, relational factors are understood as central mediating elements between the organizational context and individual intentions. The quality of relationships with colleagues and leadership figures, team cohesion, as well as the perceived support and professional recognition, directly contribute to meeting the need for relatedness [33,34,36]. A particularly significant finding concerns the role of self-efficacy as an individual factor in explaining the intention to stay. Nurses who perceive themselves as possessing the skills needed to meet clinical and organizational demands are more likely to express the intention to stay in their position or work setting [24,27,39]. In line with the Theory of Planned Behavior (TPB), self-efficacy can be conceptualized as a component of perceived behavioral control, exerting a direct influence on the formation of intention [14]. At the same time, from the perspective of SDT, it contributes to the satisfaction of the need for competence, supporting a more autonomous and resilient form of motivation [12,13]. Taken together, these results suggest that self-efficacy represents a crucial connecting node between personal resources and the intention to stay. From this perspective, job satisfaction, which emerged as a relevant variable in the included studies, can also be interpreted considering the TPB: indeed, job satisfaction contributes to the formation of a favorable attitude toward the behavior of staying, directly influencing the intention to stay [24]. The findings of this review are consistent with the international literature, which describes nurse retention as a complex, multifactorial phenomenon that is highly sensitive to the characteristics of the organizational context [7,10,47]. Previous studies have highlighted the crucial role of the work environment, supportive leadership, and job satisfaction as determinants of the intention to stay in the role [5,10]. However, a substantial portion of the available research has examined these dimensions in a fragmentary way, predominantly adopting sector-specific approaches or focusing on individual variables [5,6,8]. In high-intensity care settings, such as emergency departments and intensive care units, the international literature shows that heavy workloads, emotional pressure, and an increased risk of burnout heighten vulnerability to turnover, making retention particularly dependent on organizational support and individual resilience resources [29,32,36,39,44]. From this perspective, the post-pandemic context outlined by recent studies suggests that retention can no longer be interpreted solely as the outcome of contractual or environmental conditions but should instead be regarded as an indicator of the systemic sustainability of healthcare organizations [3,39,42]. This review contributes to broadening this perspective by systematically integrating organizational, relational, and individual factors within a shared theoretical framework. In particular, the explicit inclusion of individual psychological resources, such as self-efficacy and psychological capital, makes it possible to move beyond exclusively structural models of retention and to recognize the active role of nurses in the decision-making processes underlying the choice to remain in the workplace. Although the included studies span diverse geographical and organizational contexts, differences in healthcare systems, workforce policies, and institutional structures may influence how retention determinants manifest across settings. Therefore, while the findings provide a comprehensive synthesis of the main factors associated with nurse retention, their applicability may vary depending on the characteristics of specific healthcare systems and organizational environments.

### 4.1. Strengths and Limitations

This review presents several methodological and conceptual strengths. First, the adoption of an integrative approach made it possible to include quantitative studies, qualitative studies, and reviews, offering a broad and multidimensional understanding of the determinants of nurse retention. Second, the multilevel analysis, which distinguishes between the perspective of managers and that of frontline nursing staff, constitutes an original contribution, as it makes it possible to integrate organizational, relational, and individual factors into a single conceptual framework. A further strength lies in the explicit integration with well-established theoretical models, which has made it possible to move beyond a purely descriptive reading of the results and to propose a theoretically grounded interpretation of the mechanisms underlying nurse retention, including in the post-pandemic context. However, some limitations must be considered when interpreting the results. Most of the included studies used cross-sectional designs, which do not allow for the definitive establishment of causal relationships between the identified factors and actual retention in the role or within the organization. Furthermore, the heterogeneity of the geographical, healthcare system, and organizational contexts analyzed may limit the generalizability of the findings, although such variability also represents a strength in terms of interpretative breadth and depth. Finally, retention was frequently operationalized through intention to stay, which, although widely used and validated in the literature, does not necessarily correspond to the observable behavior of long-term retention.

### 4.2. Implications for Practice

The findings highlight how nurse retention requires the adoption of integrated strategies capable of acting synergistically on the organizational environment, on professional relational dynamics, and on the individual resources of healthcare staff. Interventions limited solely to improving structural conditions are in fact insufficient if they are not accompanied by measures aimed at strengthening intrinsic motivation, sense of organizational belonging, and perceived control over one’s work role. Healthcare organizations are therefore called upon to promote sustainable work environments and to invest systematically in training managers in transformational leadership, to enhance the levels of support, recognition, and professional development perceived by nursing staff. At the same time, programs aimed at strengthening the self-efficacy and psychological well-being of staff can significantly contribute to consolidating their intention to remain in service, especially in settings characterized by high care complexity. From this perspective, nurse retention should be conceptualized and monitored as a strategic indicator of organizational quality and of the overall sustainability of the healthcare system.

## 5. Conclusions

Nurse retention emerges from this review as a complex, dynamic, and multi-level phenomenon shaped by the interaction between organizational conditions, the quality of professional relationships, and individual psychological resources. The integration of Herzberg’s theory, Self-Determination Theory, and the Theory of Planned Behavior enables the interpretation of retention not merely as a structural outcome, but as the expression of a complex motivational and decision-making process. The distinction between the managers’ perspective and that of frontline staff represents a further contribution of the review, highlighting how retention arises from interdependent processes that are activated and interact simultaneously at different levels of healthcare systems. In light of the global challenges associated with the shortage of nursing staff and the long-term effects of the pandemic, the promotion of sustainable, safe work environments oriented toward professional well-being is a strategic priority to ensure the quality of care and the stability of the nursing workforce. Further primary studies are needed that explicitly take the multifactorial nature of the phenomenon as the main object of investigation, adopting robust theoretical frameworks to support the design and interpretation of research.

## Figures and Tables

**Table 1:** Main determinants of nurse retention.

Perspective	Factor	Key Determinants Identified
Nurse managers	Organizational	Transformational leadership, participative leadership, supportive organizational climate
	Relational	Quality of manager–staff relationships, trust, clarity of expectations
	Individual	Leadership competencies, ability to communicate vision, recognition of staff needs
Staff nurses	Organizational	Favorable work environment, adequate staffing, managerial support, professional development opportunities
	Relational	Peer support, teamwork, trust in leadership, communication climate
	Individual	Self-efficacy, resilience, psychological capital, job satisfaction, professional meaning

## Data Availability

No new data were created or analyzed in this study. Data sharing is not applicable.

## Supplementary Materials

The following supporting information can be downloaded at: https://www.mdpi.com/article/10.3390/healthcare14060772/s1, Table S1: Search strategy; Table S2. (a): JBI Critical Appraisal Checklist for Analytical Cross-Sectional Studies; (b): QuADS Quality Appraisal for Diverse Studies; Table S3: Data Extraction.

## Abbreviations

The following abbreviations are used in this manuscript:

SDT	Self Determination Theory
TPB	Theory of Planned Behavior
