# Supplementary material for: Nurse Retention in Hospitals: A Multilevel Integrative Review of Organizational Determinants
Source: Healthcare (Basel). 2026 Mar 19;14(6):772. doi: 10.3390/healthcare14060772 (PMC13026066; doi:10.3390/healthcare14060772)
Supplement: Supplementary file 1 [file healthcare-14-00772-s001.zip › healthcare-4183383-supplementary.pdf]

Table S1: Search strategy

Database	Search strategy
**Pubmed**	("personnel retention"[Title/Abstract] OR "staff retention"[Title/Abstract] OR "personnel turnover"[MeSH Terms]) AND ("working conditions"[Title/Abstract] OR "workplace"[Title/Abstract] OR "workplace"[MeSH Terms] OR (("environ"[All Fields] OR "environment"[MeSH Terms] OR "environment"[All Fields] OR "environments"[All Fields] OR "environment s"[All Fields] OR "environs"[All Fields]) AND "work"[MeSH Terms])) AND ("healthcare"[Title/Abstract] OR "health care"[Title/Abstract] OR "hospital"[Title/Abstract] OR "health services"[Title/Abstract] OR "health services"[MeSH Terms] OR "hospitals"[MeSH Terms])
**Cinahl/ApaPsycArtycles/ApaPsycInfo**	(MH "Personnel Retention" OR AB "personnel retention" OR AB "staff retention") AND (MH "Work Environment" OR MH "Workplace" OR AB "working conditions" OR AB "workplace") AND (MH "Health Facilities" OR MH "Hospitals" OR MH "Health Services" OR AB "healthcare" OR AB "health care" OR AB "hospital" OR AB "health services" OR AB "health facilities")
**Scopus**	(TITLE-ABS-KEY ("personnel retention" OR "staff retention" OR "nurse retention" OR "employee retention")) AND (TITLE-ABS-KEY ("working conditions" OR "workplace" OR "work environment")) AND (TITLE-ABS-KEY ("healthcare" OR "health care" OR "hospital" OR "health services" OR "health facilities"))

**Cochrane**	(personnel retention OR staff retention) AND (working conditions OR workplace) AND (nurses OR nursing OR healthcare professionals OR hospital)

Table S2a: JBI Critical Appraisal Checklist for Analytical Cross-Sectional Studies

Study	Were the criteria for inclusion in the sample clearly defined?	Were the study subjects and the setting described in detail?	Was the exposure measured in a valid and reliable way?	Were objective, standard criteria used for measurement of the condition?	Were confounding factors identified?	Were strategies to deal with confounding factors stated?	Were the outcomes measured in a valid and reliable way?	Was appropriate statistical analysis used?	Score
**Adalin et al. (2025)**	Yes	Yes	Yes	Yes	No	No	Yes	Yes	**6/8**
**Al-Hamdan (2016)**	Yes	Yes	Yes	Yes	Yes	Yes	Yes	Yes	**8/8**
**Alshaibani (2024)**	Yes	Yes	Yes	Yes	No	No	Yes	Yes	**6/8**
**Ashwini e Padhy (2024)**	No	Yes	Yes	Yes	No	No	Yes	Yes	**5/8**
**Blegen et al. (2017)**	Yes	Yes	Yes	Yes	Yes	Yes	Yes	Yes	**8/8**
**Chua et al. (2024)**	Yes	Yes	Yes	Yes	Yes	Yes	Yes	Yes	**8/8**
**Eltaybani et al. (2018)**	Yes	Yes	Yes	Yes	Yes	Yes	Yes	Yes	**8/8**
**Hignight et al. (2024)**	Yes	Yes	No	Yes	No	No	Yes	Yes	**5/8**

**Hossny et al. (2023)**	Yes	Yes	Yes	Yes	Yes	Yes	Yes	Yes	**8/8**
**Kim e Sook Yoo (2018)**	Yes	Yes	Yes	Yes	Yes	Yes	Yes	Yes	**8/8**
**Jin et al. (2024)**	Yes	Yes	Yes	Yes	Yes	Yes	Yes	Yes	**8/8**
**Kao e Kao (2024)**	No	Yes	Yes	Yes	Yes	Yes	Yes	Yes	**7/8**
**Mulkey e Casey (2023)**	Yes	Yes	Yes	No	No	No	Yes	Yes	**5/8**
**Ofei e Paarima (2022)**	Yes	Yes	Yes	Yes	Yes	Yes	Yes	Yes	**8/8**

Table S2b: QuADS Quality Appraisal for Diverse Studies (Harrison, 2021)*

**QuADS** *Quality Appraisal for diverse studies*	**Theoretical or conceptual underpinning to the research**	**Statement of research aim/s**	**Clear descriptions of research setting and target population**	**The study design is appropriate to address the stated research aim/s**	**Appropriate sampling to address the research aim/s**	**Rationale for choice of data collection tool/s**	**The format and content of data collection tool is appropriate to address the stated research aim/s**	**Description of data collection procedure**	**Recruitment data provided**	**Justification for analytic method selected**	**The method of analysis was appropriate to answer the research aim/s**	**Evidence that the research stakeholders have been considered in research design or conduct.**	**Strengths and limitations critically discussed**	**Quality scores**
**Arakelian et al. (2019)**	**3**	**3**	**3**	**3**	**3**	**3**	**3**	**3**	**3**	**3**	**3**	**2**	**3**	**38**
**Ejebu et al. (2025)**	**3**	**3**	**3**	**3**	**3**	**3**	**3**	**3**	**3**	**3**	**3**	**3**	**3**	**39**
**Ingerslev Loft e Jensen (2020)**	**3**	**3**	**3**	**3**	**3**	**3**	**3**	**3**	**3**	**3**	**3**	**2**	**3**	**38**

**Kilcommons et al. (2026)**	**3**	**3**	**3**	**3**	**3**	**3**	**3**	**3**	**3**	**3**	**3**	**2**	**3**	**38**
**Spoolder et al. (2024)**	**3**	**3**	**3**	**3**	**2**	**3**	**3**	**3**	**3**	**3**	**3**	**2**	**3**	**37**
**Washeya e Fürst (2021)**	**3**	**3**	**3**	**3**	**3**	**2**	**3**	**3**	**3**	**3**	**3**	**2**	**3**	**37**
**Farahani et al. (2024)**	**3**	**3**	**3**	**3**	**3**	**2**	**3**	**3**	**3**	**3**	**3**	**0**	**3**	**34**
**Goens e Giannotti (2024)**	**3**	**3**	**2**	**3**	**2**	**2**	**3**	**3**	**3**	**2**	**3**	**0**	**3**	**32**
**McIntyre et al. (2024)**	**2**	**3**	**3**	**3**	**3**	**2**	**3**	**2**	**3**	**3**	**3**	**0**	**3**	**32**
**Phakdeechanuan et al. (2024)**	**3**	**3**	**3**	**3**	**3**	**3**	**3**	**3**	**3**	**3**	**3**	**2**	**3**	**38**
**Pressley e Garside 82022)**	**3**	**3**	**2**	**3**	**3**	**2**	**3**	**3**	**3**	**3**	**3**	**0**	**3**	**34**

*Rationale for using QuADS on reviews: QuADS items were adapted to the review context: sampling ↦ search/selection strategy; data collection ↦ data extraction/charting; analysis ↦ narrative synthesis / meta-analysis. This adaptation was pre-specified to ensure consistent appraisal across heterogeneous designs

Table S3: Data extraction

**N°**	**Authors (Year of publication)**	**Title**	**Study Type and Aim**	**Sample and setting**	**Main Results**	**Declared Limitations**	**JBI**
**1**	**Adalin et al. (2025)**	Influence of Transformational Leadership Competence on Nurses' Intent to Stay: Cross-Sectional Study	Quantitative –cross-sectional study<br><br>**Aim:** to examine the relationship between nurse managers' transformational leadership (overall and dimensions) and staff nurses' intent to stay, and at identifying whether transformational	A total of 523 registered nurses providing direct patient care were recruited using probabilistic cluster sampling from three hospitals within an academic medical city (King Saud University Medical City) in Riyadh, Saudi Arabia. Participants had at	Nurse managers' transformational leadership was positively and significantly associated with nurses' intent to stay ($r = 0.22$, $p < 0.001$). All five transformational leadership dimensions showed significant positive correlations with intent to stay. Multivariable ridge regression indicated that overall transformational leadership was a significant predictor of nurses' intent to stay ($\beta = 0.13$,	The study relied on self-reported, cross-sectional data, limiting causal inference and introducing potential self-selection and social desirability bias.<br><br>Intent to stay was measured as an attitudinal outcome rather than actual retention.<br><br>The regression model accounted for a limited proportion of variance and did	**Quality Rating: Moderate**

			leadership predicts intent to stay.	least one year of employment and represented multiple clinical units.	p < 0.001), whereas individual leadership dimensions were not independently predictive.	not include all potential confounding variables.<br><br>Findings may not be generalizable beyond the specific organizational and cultural context studied.	
**2**	**Al-Hamdan et al. (2016)**	Jordanian Nursing Work Environments, Intent to Stay, and Job Satisfaction	Quantitative –cross-sectional study<br><br>**Aim:** to examine the associations among the nursing work environment, job satisfaction, and nurses' intent to stay in hospital settings in Jordan.	A convenience sample of 582 registered nurses working on inpatient units in three hospitals in Jordan Inclusion criteria were registered nurses with at least one year of clinical experience.<br><br>Data were collected using validated self-report instruments (PES-NWI, McCain Intent to Stay scale, Global Job Satisfaction survey).	The nursing work environment was positively associated with both job satisfaction and intent to stay. For each one-unit increase in the PES-NWI score, job satisfaction increased by 1.3 points (p < .001) and intent to stay increased by 3.6 points (p < .001), after adjustment for hospital and unit type. Nurses working in public hospitals reported significantly higher job satisfaction and intent to stay compared with those in teaching hospitals. Unit type was associated with job satisfaction but not with intent to stay.	The cross-sectional design precludes causal inference.<br><br>All measures were self-reported and may not reflect objective work environment characteristics.<br><br>The sample was drawn from hospitals in only two cities, limiting generalizability to other regions or healthcare contexts.	**Quality Rating: High**
**3**	**Alshaibani et al. (2024)**	Association of Job Satisfaction, Intention to Stay, Organizational Commitment, and General Self-Efficacy Among Clinical Nurses in Riyadh, Saudi Arabia	Quantitative –cross-sectional study<br><br>**Aim:** to explore the relationships among job satisfaction, intention to stay, organizational commitment, general self-efficacy, and demographic variables.	A convenience sample of 227 clinical nurses working in a major referral hospital providing secondary and tertiary care in Riyadh, Saudi Arabia. Participants included registered nurses, licensed practical nurses, and nurse managers with at least six months of employment.	Moderate levels of job satisfaction, intention to stay, organizational commitment, and self-efficacy were reported. Significant positive correlations were found between job satisfaction and intention to stay (r = 0.362, p < 0.01), organizational commitment (r = 0.657, p < 0.01), and self-efficacy (r = 0.578, p < 0.01). Intention to stay was positively associated with organizational commitment (r = 0.486, p < 0.01) and self-efficacy (r = 0.489, p < 0.01).	The cross-sectional design limits causal inference.<br><br>Data were self-reported, introducing potential response and social desirability bias.<br><br>Findings are context-specific to a single hospital and may not be generalizable to other healthcare settings.	**Quality Rating: Moderate**

				Data were collected using validated measurement tools, including the Job Satisfaction Index, the Intent to Stay Scale, the Organizational Commitment Scale, and the General Self-Efficacy Scale, were utilized.			
**4**	**Ashwini e Padhy (2024)**	Development of a Nursing Workforce: Influence of workplace spirituality, health support, extrinsic rewards, and professional commitment on nurse retention	Quantitative –cross-sectional study<br><br>**Aim:** to examine the direct and mediating effects of professional commitment, workplace support for health, intrinsic job satisfaction, extrinsic rewards, and workplace spirituality on nurse retention, and at testing a conceptual mediation model of nurse retention.	A convenience sample of 396 clinical nurses working in five hospitals in the Vellore region, India.<br><br>Data were collected using self-administered questionnaires adapted from validated instruments.	Extrinsic rewards, professional commitment, and workplace support for health showed significant positive direct effects on nurse retention. Intrinsic job satisfaction did not have a significant direct effect on retention but demonstrated a significant indirect effect through workplace spirituality. Workplace spirituality significantly mediated the relationships between extrinsic rewards, intrinsic job satisfaction, and professional commitment with nurse retention, but did not mediate the relationship between workplace support for health and retention. The structural model demonstrated high explanatory power for nurse retention ($R^2 = 0.910$).	The cross-sectional design limits causal inference.<br><br>The use of convenience sampling and self-reported measures introduces potential response and common-method bias.<br><br>The study was conducted in a single geographic region, limiting generalizability to other healthcare contexts.<br><br>Longitudinal and experimental designs were recommended for future research.	**Quality Rating: Moderate**
**5**	**Blegen et al. (2017)**	Newly Licensed RN Retention	Quantitative observational study (secondary analysis of longitudinal data)<br><br>**Aim:** to examine 1-year retention of newly licensed registered nurses	Secondary analysis of data from 1,464 newly licensed RNs employed across 97 hospitals in three U.S. states (Illinois, North Carolina, Ohio). Hospitals varied by size,	The overall 1-year retention rate was 83%. Retention was significantly higher in Magnet hospitals (92%) compared with non-Magnet hospitals (77%), in urban hospitals, in larger hospitals, and in university-affiliated hospitals. Hospital characteristics had a stronger	Retention was assessed only during the first year of employment, limiting insight into longer-term retention.<br><br>Hospitals participated voluntarily in the parent study,	**Quality Rating: High**

			(NLRNs) employed in hospitals and identifying hospital and nurse characteristics most strongly associated with retention.	urban/rural location, Magnet designation, ownership, and university affiliation. Nurse-level data were available for a subsample of 1,082 respondents.	influence on retention than individual nurse characteristics. Among nurse variables, age was the only factor significantly associated with retention, with younger nurses more likely to stay.	which may limit representativeness.<br><br>Findings may not generalize to all U.S. hospital settings despite the diversity of hospitals and nurses included.	
**6**	**Chua et al. (2024)**	Personal and Workplace Characteristics as Predictors of Intent-To-Stay Among Registered Nurses: An Exploratory Quantitative Multicentre Study	Quantitative –cross-sectional study<br><br>**Aim:** to describe and examine the factors associated with registered nurses' intent-to-stay and subsequently identify predictors of nurses' intent-to-stay.	A convenience sample of 270 registered nurses working across multiple healthcare institutions in Singapore.<br><br>Participants were registered nurses aged ≥21 years, recruited via institutional channels and social media, and completed an online questionnaire.	Intent-to-stay levels were moderate (mean = 2.96). Significant positive correlations were found between intent-to-stay and resilience, occupational self-efficacy, self-realisation, and workload, while sleep disturbance was negatively correlated with intent-to-stay. Multivariable regression analyses identified occupational self-efficacy, workload, nurses' designation, and specialisation status as significant predictors of intent-to-stay, collectively explaining approximately 10–11% of the variance.	The cross-sectional design precludes causal inference.<br><br>Convenience sampling limits generalisability.<br><br>Data were self-reported, introducing potential common-method bias.<br><br>The regression models explained a relatively small proportion of variance, suggesting the presence of additional unmeasured determinants of intent-to-stay.	**Quality Rating: High**
**7**	**Eltaybani et al. (2018)**	Factors related to intention to stay in the current workplace among long- term care nurses: A nationwide survey	Quantitative- A cross-sectional questionnaire survey.<br><br>**Aim:** To identify the factors associated with long-term care nurses' intention to stay in their current workplace.	A total of 3128 staff nurses and 257 nurse managers from the long-term care wards of the participating hospitals.	Only 40.1% of nurses reported an intention to stay. Multilevel logistic regression showed that intention to stay was positively associated with nurses' age, years of employment in the current hospital, previous workplace changes, and specific reasons for choosing the workplace. At the individual level, higher work engagement was positively associated with intention to stay, whereas higher burnout was negatively associated. At the unit level, receiving appropriate support from nurse	The cross-sectional design precludes causal inference.<br><br>Despite nationwide coverage and large sample size, the hospital participation rate was low, potentially affecting representativeness.<br><br>Some relevant constructs (e.g., organizational commitment and job satisfaction) were not included.<br><br>Psychometric properties of certain measurement scales	**Quality Rating: High**

					managers, higher perceived quality of the care process, and greater educational opportunities were significant positive predictors. Nurses' qualifications, unit size, and patients' medical acuity were not associated with intention to stay.	were not fully assessed, and findings may not generalize beyond the Japanese long-term care hospital context.	
8	**Hignight et al. (2024)**	Development of an emergency nurse internship to promote a healthy work environment and improve nurse retention	Quantitative - Evidence-based practice quality improvement (EBPQI) pre–post intervention study<br><br>**Aim:** to evaluate the impact of a structured, competency-based emergency nurse internship program on new emergency nurse retention and on the health of the work environment.	The study was conducted in a 33-bed community emergency department in the Midwestern United States. The pre-intervention cohort included 22 emergency nurses hired between January 2021 and January 2022 (new graduate nurses and nurses new to the ED), while the post-intervention cohort included 9 emergency nurses hired between February 2022 and January 2023. The intervention consisted of a 16-week emergency nurse internship implemented for all new-to-ED nurses.	Nurse turnover decreased significantly following implementation of the internship program, with total turnover declining from 81.8% pre-intervention to 11.1% post-intervention ($p < .001$). Retention improved significantly for both new graduate nurses and nurses new to the emergency department. In addition, scores on the AACN Healthy Work Environment Assessment Tool increased significantly overall (mean score from 3.15 to 3.77, $p < .001$), with significant improvements observed in skilled communication, appropriate staffing, meaningful recognition, and authentic leadership	The initiative was conducted in a single emergency department with a small sample size, limiting generalisability.<br><br>Multiple interventions aimed at improving the work environment were implemented concurrently, making it difficult to isolate the specific effect of the internship program.<br><br>As a quality improvement initiative, findings are context-specific and not intended to be generalisable beyond the study setting.	**Quality Rating: Moderate**
9	**Hossny et al. (2023)**	Influence of nurses' perception of organizational climate and toxic leadership behaviors on	Quantitative- a descriptive comparative study<br><br>**Aim**: to assess nurses' perceptions of organizational	A sample of 250 nurses selected through simple random sampling from two large hospitals in Assiut,	Most nurses reported a "normal" level of intent to stay, with a substantial proportion indicating low intention to stay, particularly in the insurance hospital. Nurses perceived the organizational climate as	The study was conducted in only two hospitals within one Egyptian city, limiting generalizability.<br><br>Sample sizes differed between hospital types. The sample	**Quality Rating: High**

		intent to stay: A descriptive comparative study	climate and toxic leadership behaviors, examining their relationships with nurses' intent to stay, and comparing these variables between a university hospital and an insurance hospital.	Egypt: one university hospital (n = 150) and one health insurance hospital (n = 100).<br><br>Data were collected using validated self-administered questionnaires.	generally poor, while toxic leadership behaviors were perceived at a low to moderate level. Multivariate regression analyses showed that supportive systems and performance standards were the strongest organizational climate predictors of intent to stay. Regarding toxic leadership, authoritarian leadership and unpredictability significantly reduced intent to stay in the university hospital, whereas self-promotional leadership behaviors were the main negative predictor in the insurance hospital. Significant differences in organizational climate, toxic leadership, and intent to stay were observed between the two hospital types.	included predominantly female nurses and excluded nurses with bachelor's degrees, restricting representativeness.<br><br>The cross-sectional design and reliance on self-reported data limit causal inference.	
**10**	**Jim e Yoo (2018)**	The influence of psychological capital and work engagement on intention to remain of new graduate nurses	Quantitative- cross-sectional survey<br><br>**Aim:** to examine the influence of psychological capital (PsyCap) and work engagement (WE) on new graduate nurses' intention to remain in nursing, after controlling for selected general characteristics.	A sample of 156 new graduate nurses working in two tertiary university hospitals and one university hospital in South Korea.<br><br>Data were collected using structured self-administered questionnaires.	New graduate nurses' intention to remain was significantly and positively correlated with psychological capital ($r = 0.511$, $p < .001$) and work engagement ($r = 0.497$, $p < .001$). Hierarchical regression analysis showed that psychological capital ($\beta = 0.376$, $p < .001$) and work engagement ($\beta = 0.283$, $p = .001$) were significant predictors of intention to remain, jointly explaining 33.5% of the variance, after controlling for clinical experience, educational background, and working in a desired unit.	The regression model explained a limited proportion of variance, indicating that additional unmeasured factors may influence intention to remain.<br><br>Work context variables were not directly measured.<br><br>The cross-sectional design prevents causal inference, and potential mediating or moderating relationships were not examined.<br><br>Findings are specific to the Korean hospital context and may not be generalizable to other settings.	**Quality Rating: High**

**11**	**Jin et al. (2024)**	Association between psychological empowerment and intent to stay among military hospital nurses: the mediating effects of the practice environment and burnout	Quantitative- a cross-sectional survey<br>**Aim: t**o explore the impact of psychological empowerment on nurses' intent to stay in military hospitals as well as the mediat- ing effects of the practice environment and burnout in this context.	A convenience sample of 1,225 nurses recruited from nine military hospitals in Shaanxi Province, China.<br>Data were collected via an anonymous online questionnaire platform.	Psychological empowerment was positively and significantly associated with intent to stay ($\beta = 0.396$, $p < .001$). Mediation analyses showed that the practice environment, burnout, and their chain mediating effect partially mediated this relationship. The practice environment accounted for the largest proportion of the total effect (54.5%), followed by burnout (2.8%) and the sequential pathway practice environment and burnout (1.5%). Nurses reporting higher psychological empowerment perceived a better practice environment, experienced lower burnout, and demonstrated higher intent to stay.	The cross-sectional design limits causal inference.<br>Data were self-reported, increasing the risk of response and common-method bias.<br>The study did not explore the differential effects of the individual dimensions of psychological empowerment.<br>Findings are context-specific to Chinese military hospitals and may not be generalisable to civilian or non-Chinese settings.	**Quality Rating: High**
**12**	**Kao e Kao (2024)**	Why and how the interpersonal stressors influence nurses 'intention to stay and job satisfaction: the JD-R model perspective	Quantitative- cross-sectional survey study grounded in the Job Demands–Resources (JD-R) model.<br>**Aim:** to examine how interpersonal stressors and interpersonal resources influence nurses' intention to stay and job satisfaction, through the mediating roles of emotional exhaustion and work engagement, and the moderating roles of gratitude	A convenience sample of 288 hospital nurses working in a regional hospital in Taiwan.<br>Data were collected using validated self-administered questionnaires distributed in two waves, six months apart.	Interpersonal stressors were positively associated with emotional exhaustion and negatively associated with job satisfaction and intention to stay, with emotional exhaustion fully mediating these relationships. Interpersonal resources were positively associated with work engagement, which in turn partially mediated their effects on both job satisfaction and intention to stay. Awareness of employee assistance programs moderated the relationship between interpersonal resources and work engagement, showing a substitution effect. Gratitude did not significantly moderate the effects of interpersonal stressors on emotional exhaustion or retention	The study employed a cross-sectional design, limiting causal inference despite the two-wave data collection.<br>Participants were recruited from a single hospital, restricting generalisability.<br>The focus on overall interpersonal relationships did not distinguish between different relational sources (e.g., supervisors vs. peers).<br>Findings may not be transferable beyond the East Asian hospital context.	**Quality Rating: High**

			and employee assistance programs (EAPs).		outcomes. The findings supported the JD-R health impairment and motivational pathways in explaining nurse retention.		
13	**Mulkey e Casey (2023)**	Factors that influence the retention of graduate nurses at a safety net teaching hospital	Quantitative study<br><br>**Aim:** to determinate retention and program completion rates of new graduate registered nurses (NGRNs) enrolled in a nurse residency program (NRP);<br>to identify perceived work environment, support, encouragement, and reward factors influencing retention among NGRNs with more than one year of experience.	The study was conducted in a 555-bed urban, level I trauma, safety-net teaching hospital in the United States.<br><br>Phase 1 included 429 NGRNs enrolled in the NRP for retention and completion analyses.<br><br>Phase 2 involved 80 former NGRNs with more than one year of experience who completed the Casey–Fink Nurse Retention Survey to explore perceived retention factors.	The average NRP completion rate over seven years was 95%. Retention rates were 93% at 1 year, 82% at 2 years, 74% at 3 years, 66% at 4 years, and 59% at 5 years. Survey findings indicated that recognition and rewards, supportive leadership, positive professional nursing role perceptions, and mentorship-related factors were perceived as key influences on retention. Nurses valued feeling respected, supported by charge nurses and managers, and encouraged in professional development, while scheduling flexibility was rated as the least influential factor.	The project was conducted in a single safety-net teaching hospital, limiting generalisability.<br><br>Survey response rate was low, introducing potential response bias.<br><br>Retention factors were assessed only among nurses who remained employed, excluding perspectives of those who had left. As a quality improvement initiative, the study lacked a control group and causal relationships cannot be inferred.	**Quality Rating: Moderate**
14	**Ofei e Paarima (2022)**	Nurse managers leadership styles and intention to stay among nurses at the unit in Ghana	Quantitative- cross sectional study<br><br>Aim: to describe nurse managers' leadership styles and examining their influence on nurses' intention to stay at the unit level in hospital settings.	A sample of 348 nurses recruited from 38 hospitals in Ghana, including primary (district), secondary (regional), and tertiary hospitals. Participants included registered nurses, midwives, and enrolled nurses who had worked for at least one year in the hospital and at least	Nurses reported a high overall intention to stay (mean = 3.26/4), with 85.6% indicating no intention to leave their current workplace and 82.8% not planning to leave within the next 12 months. Nurse managers most frequently used participative leadership, followed by transformational leadership, while laissez-faire leadership was least used. Transformational ($r = 0.326$, $p < .001$) and participative leadership styles ($r = 0.226$, $p < .001$) were positively associated with intention to	The cross-sectional design limits causal inference.<br><br>Data were self-reported, raising the possibility of response bias.<br><br>Hospitals were purposefully selected and the sample was limited to Ghanaian public and faith-based hospitals, which may restrict generalisability to other healthcare systems or cultural contexts.	**Quality Rating: High**

				three months with their nurse manager.<br><br>Data were collected using Multifactor Leadership Questionnaire (MLQ-5x)	stay, whereas transactional, autocratic, and laissez-faire leadership styles were negatively associated. Multivariate regression showed that leadership styles jointly explained 20.9% of the variance in intention to stay, with transformational leadership positively predicting intention to stay, and transactional and autocratic leadership exerting significant negative effects.		
**15**	**Arakelian et al. (2019)**	I stay—swedish specialist nurses in the perioperative context and their reasons to stay at their workplace	Qualitative study<br><br>**Aim:** To investigate why nurse anesthetists and operating room nurses choose to stay in the same workplace	A purposive sample of 15 specialist nurses (7 nurse anesthetists and 8 operating room nurses) recruited from four hospitals in Sweden. All participants had worked continuously in the same perioperative department for at least five years.	Three main themes explained nurses' decisions to stay:<br><br>1)**Organizational stability and homelikeness**, characterized by low staff turnover, non-hierarchical culture, collegial support, and a sense of belonging;<br>2) **Sustained professional development,** with opportunities for role variation, learning, and use of advanced clinical expertise over time;<br>3) **Humane and facilitative leadership**, where head nurses were visible, supportive, flexible, and enabled work–life balance and professional growth.<br><br>Together, these factors created a welcoming, stable work environment that fostered long-term retention.	The study included a small sample and predominantly older specialist nurses, potentially limiting transferability to younger nurses.<br><br>Most interviews were conducted by telephone rather than face-to-face.<br><br>Fewer male nurses participated, reflecting workforce demographics but limiting gender representation.<br><br>Findings are context-specific to Swedish perioperative settings.	**Quality Rating: High**
**16**	**Ejebu et al. (2025)**	Values and workplace expectations to facilitate retention: perspectives from	Qualitative study<br><br>**Aim:** to explore what early-career (EC) and late-career (LC) NHS nurses value and expect	Qualitative component: 27 registered NHS nurses in England (11 early-career nurses registered; 16 late-	Two overarching themes explained nurse retention across career stages:<br>1) **Valuing nursing (systemic and professional factors)**, including professional identity, pride in patient care, supportive	The study focused exclusively on NHS nurses in England, limiting transferability to non-NHS or international settings.<br><br>Participants were predominantly White and	**Quality Rating: High**

		nurses at two ends of the career spectrum	from employers to remain in their jobs and in the nursing profession, and at identifying common and divergent retention factors across career stages.	career nurses aged ≥55 years) participated in 8 focus groups and 7 interviews conducted online via Microsoft Teams.<br><br>Supplementary data: 784 open-text responses from a national cross-sectional survey of NHS nurses in England (2023).	team relationships, leadership quality, organisational culture, and the ability to deliver care despite chronic understaffing and resource constraints;<br>2) **Valuing nurses (individual expectations)**, encompassing recognition of expertise, career development opportunities, flexible working patterns, work–life balance, and fair remuneration.<br>Persistent factors detrimental to retention across both EC and LC nurses included negative work culture, lack of recognition, insufficient staffing and resources, and limited career development opportunities. Leadership support and collegial relationships acted as key buffers against stressful working environments.	female, under-representing minority ethnic perspectives.<br><br>Some overlap existed between age and career-stage definitions.<br><br>Qualitative data were collected as part of a larger programme designed to inform discrete choice experiments, which may have influenced topic emphasis.	
**17**	**Ingerslev Loft et al. (2020)**	What makes experienced nurses stay in their position? A qualitative interview study	Qualitative study<br><br>**Aim:** to explore which factors are important in experienced nurses' intention to stay in the clinical setting and to learn which factors affect their job satisfaction.	A purposive sample of 28 experienced registered nurses (≥3 years in current position) recruited from six hospitals (including university and local hospitals) across two regions of Denmark. Participants represented multiple specialties (e.g., neurology, paediatrics, surgical, haemodialysis, outpatient care).<br><br>Data were collected through individual	Seven interrelated themes explained experienced nurses' intention to stay and job satisfaction:<br>1) **Being an experienced nurse**, associated with feelings of confidence, security, and professional identity;<br>2)**Importance of the speciality,** characterised by variety and sustained professional interest rather than prestige;<br>3) **Management**, particularly the pivotal role of visible and supportive charge nurses;<br>4) **Professional challenges**, including opportunities for development and meaningful use of expertise;	The study included only Danish nurses, limiting transferability to other cultural or healthcare contexts.<br><br>Although participants varied in age, experience, and specialty, findings may not represent all experienced nurses.<br><br>Qualitative transferability relies on reader judgement despite the provision of rich descriptions.	**Quality Rating: High**

				semi-structured interviews conducted between September 2018 and April 2019.	5) **Good colleagues**, fostering team cohesion and mutual support;<br>6) **Balancing family and work–life**, identified as a central determinant of retention;<br>7) **Organisational change**, which could either support or undermine retention depending on transparency, recognition, and workload impact.<br><br>Retention decisions were multifactorial and not primarily linked to specialty type.		
**18**	**Kilcommons et al. (2026)**	Exploring the factors affecting ICU nurse retention during and post-COVID-19: A qualitative descriptive interview study	Qualitative study<br><br>**Aim:** to explore identifying and exploring the factors that promoted or could improve ICU nurse retention during and after the COVID-19 pandemic.	A purposive sample of 19 registered nurses recruited from a single medical–surgical intensive care unit in a tertiary care centre in Alberta, Canada.<br><br>Participants had either left or seriously considered leaving their ICU position between January 2020 and August 2022. Interviews were conducted virtually (audio/video recorded).	Four main themes influenced nurses' decisions to remain in the ICU:<br>1) **Organizational resources and scheduling**, including adequate staffing levels, equitable workload distribution, flexible scheduling, and appropriate financial compensation;<br>2) **Interpersonal factors, particularly management responsiveness**, inclusive communication, and supportive team relationships;<br>3) **Mental health support**, encompassing access to effective psychological support, recognition, and validation of nurses' professional contributions;<br>4)**Training and career advancement, including improved ICU-specific orientation**, ongoing professional development, mentorship, and clear career progression pathways.	The study was conducted in a single ICU, limiting transferability to other settings.<br><br>Participants were predominantly female and Caucasian.<br><br>Potential response bias was present, as nurses with stronger negative experiences may have been more inclined to participate.<br><br>Perspectives of nurses who had definitively decided to stay were not included.	**Quality Rating: High**

					Participants emphasised that administrative support, mental health resources, and opportunities for growth could mitigate burnout and enhance retention.		
**19**	**Spoolder et al. (2024)**	Engaging, binding and retaining nurses: the success formula of an exemplary ward	Qualitative study<br><br>**Aim:** to identify the factors that contribute to the success of the urology ward in engaging, binding, and retaining nurses, to provide practical insights and opportunities for other wards.	A purposive sample of 12 registered nurses (all female) working on a urology ward of a non-academic teaching hospital in the Netherlands.<br><br>Nurses were divided into three focus groups based on work experience (<1 year, 1–5 years, >5 years).	Four overarching themes explained the ward's success in nurse retention:<br>1) **Supportive structures, including strong nursing governance**, continuous professional development, and high-quality, trust-based leadership;<br>2) **Optimal work environment**, characterised by skilled colleagues, strong interprofessional collaboration with physicians, and high professional autonomy;<br><br>3) **Team culture,** marked by feeling seen, equality, psychological safety, and a positive social atmosphere;<br>4) **Specialty of urology,** offering varied work, a manageable and predictable workload, and a patient population perceived as rewarding to care for.<br><br>Together, these factors met both nurses' professional and personal needs, fostering high job satisfaction and sustained retention.	The study was conducted in a single ward within one Dutch hospital, limiting transferability.<br><br>All participants were female and relatively young, although this reflected the ward's workforce composition.<br><br>Focus groups were purposively sampled, which may have introduced selection bias.<br><br>Findings are context-specific and rely on qualitative transferability rather than generalisability.	**Quality Rating: High**
**20**	**Washeya e Fürst (2021)**	Work features that influence the retention of professional nurses in the public health sector in	Qualitative study<br><br>**Aim:** to explore professional nurses' perceptions of factors influencing retention in the	A purposive sample of 11 professional nurses (10 female, 1 male; aged 25–56 years, with 4–33 years of experience), including staff nurses	Two overarching themes with six categories were identified:<br>1) **Satisfaction with remuneration varied**; the work environment was non-conducive and management was inadequate (hygiene	The study was conducted in one public hospital, limiting transferability to other settings.	**Quality Rating: High**

		windhoek, namibia	public health sector in Windhoek, Namibia.	and nurse managers, recruited from one major public training hospital in Windhoek, Namibia.<br><br>Data were collected through semi-structured, face-to-face interviews conducted in hospital and home settings.	factors), encompassing remuneration and allowances, poor physical work environment (staff shortages, inadequate equipment, overcrowding), and insufficient management support and supervision;<br>2) **Dissatisfaction due to negative psychological effects of the work environment and lack of career development opportunities** (motivation factors), including stress, guilt, frustration, workplace violence, feeling unsafe and uncared for, and perceived bias and unfairness in access to study leave and professional development.	The small sample size and context-specific findings restrict generalisability.<br><br>As with all qualitative research, findings rely on participants' subjective accounts and reader judgement for transferability.	
**21**	**Farahani et al. (2024)**	Factors affecting nurses retention during the COVID-19 pandemic: a systematic review	Systematic review<br><br>**Aim:** to explore factors affecting retention of nurses.	A total of 18 primary conducted across multiple countries.<br><br>Studies were retrieved from PubMed, Scopus, Web of Science, ProQuest, and Google Scholar, without time limitation, and included nurses working in various healthcare settings during the COVID-19 pandemic.	Seven broad categories of factors influencing nurse retention during the COVID-19 pandemic were identified:<br>1) **Personal factors** (e.g., demographic characteristics, personal values, resilience, professional commitment);<br>2) **Interpersonal factors** (e.g., teamwork, collegial support, communication, nurse–patient relationships);<br>3) **Organizational factors** (e.g., leadership support, staffing and workload, work environment, organizational commitment, financial and non-financial incentives);<br>4) **Social media factors** (e.g., public recognition of nursing, media portrayal of the pandemic);<br>5) **Educational factors** (e.g., COVID-19–related training,	The review focused exclusively on studies related to the COVID-19 pandemic, limiting transferability to non-pandemic contexts.<br><br>Included studies were heterogeneous in design, measures, and quality.<br><br>Although quality appraisal was conducted using JBI tools, the overall strength of evidence varied.<br><br>Only English-language studies were included, introducing potential language bias.	**Quality Rating: High**

					preparedness education, continuous professional development);<br>6) **Emotional factors** (e.g., stress, burnout, well-being, psychological preparedness);<br>7) **Protective factors** (e.g., access to personal protective equipment, staff safety measures, testing and infection control).<br><br>Overall, nurse retention during the pandemic was shown to be complex and multifactorial, influenced by factors ranging from individual to macro-organizational levels.		
**22**	**Goens e Giannotti (2024)**	Transformational leadership and nursing retention: an integrative review	Integrative review<br><br>**Aim:** to establish current evidence on the relationship between transformational nursing leadership and turnover intention.	The review included 18 studies published between 1992 and 2022.<br>Studies were conducted across multiple countries.<br><br>Nurses worked primarily in hospital settings, including public, private, teaching, and university-affiliated hospitals.<br>Literature was retrieved from CINAHL, MEDLINE (Ovid), PubMed, and ProQuest.	The review found mixed evidence regarding the direct effect of transformational leadership on nurses' intention to stay or turnover intention. While some studies demonstrated significant positive associations, others reported weak or non-significant relationships. However, there was consistent evidence that transformational leadership positively influences job satisfaction, organizational commitment, safety climate, and work environment, which in turn indirectly enhance nurse retention. Transformational leadership was also associated with lower job stress and burnout, and its effect on retention was often mediated by factors such as emotional labour, safety climate, emotional intelligence, and organizational commitment. Overall, transformational leadership emerged as an	The review included studies with heterogeneous designs, measures, and methodological quality, limiting comparability and synthesis strength.<br><br>Most included studies were cross-sectional, restricting causal inference. Evidence was heavily weighted toward quantitative studies, with limited qualitative insights.<br><br>Only English-language publications were included, introducing potential language bias.<br><br>The review did not conduct a meta-analysis, and conclusions rely on narrative synthesis.	**Quality Rating: High**

					important, but not standalone, contributor to nurse retention within a multifactorial framework.		
**23**	**McIntyre et al. (2024)**	Factors that contribute to turnover and retention amongst emergency department nurses: A scoping review	Scoping review<br><br>**Aim:** to synthesise the evidence and assess the scope of literature regarding factors that contribute to turnover and retention amongst emergency nurses.	A total of 31 studies were included in the review.<br><br>Five databases (Embase, MEDLINE, PsycINFO, CINAHL, and Business Source Complete) were searched for papers published in English between January 2011 and June 2023 where the population was nurses, context was the emergency department, and the concept was turnover or retention.	Two themes were identified:<br>1) **Turnover factors:** workplace violence (WPV), burnout, depression, poor organizational characteristics (e.g., lack of collaboration, weak communication), and environmental/job stressors (e.g., workload pressure, lack of engagement).<br>2) **Retention factors:** structured mentoring programs, development of advanced clinical skills, transition-to-specialty practice (TSPP) programs, positive leadership, teamwork, and interprofessional relationships.	Many included studies relied on self-reported data, introducing response bias risk.<br><br>Predominance of cross-sectional designs, limiting causal inference.Few interventional studies, often lacking control groups, reducing internal and external validity.<br><br>Review limited to English-language, peer-reviewed studies; grey literature excluded.<br>Initial screening and data extraction conducted by one author, increasing potential selection or extraction bias.	**Quality Rating: High**
**24**	**Phakdeechanuan et al. (2024)**	Addressing registered nurse retention and attrition in thailand hospitals: an integrative review	Integrative review<br><br>**Aim:** to analyse factors influencing the attrition and retention of RNs practicing in Thailand's hospital sector.	Sources for review totalled 35. The databases searched included CINAHL (via EBSCOhost), EMBASE, Nursing Allied (via ProQuest), Ovid, Scopus, Web of Science, and Medline from 2012 to 2024.	Three key themes emerged from thematic synthesis:<br>1) **Mental health and well-being:** Positive psychological states promote job satisfaction and retention; poor mental health, burnout, and emotional exhaustion drive attrition;<br>2) **Working environment and organization**: Low autonomy, hierarchical structures, and lack of support from supervisors and co-workers undermine motivation and satisfaction; supportive environments and collaborative leadership enhance retention;<br>3) **Workplace location**: Nurses prefer working near their hometown due to family	Over reliance on quantitative cross-sectional designs limited causal inference.<br><br>Underrepresentation of qualitative perspectives limits understanding of nurses' lived experiences.<br><br>Limited exploration of intervention effectiveness and economic impacts.<br><br>English and Thai language restriction; possible omission of relevant grey literature.	**Quality Rating: High**

					ties, reduced commuting time, and better work–life balance, which fosters motivation and retention;<br>4) **Identified significant gaps:** Most research focused on single-hospital sites and used quantitative descriptive methods; limited qualitative exploration of "how" daily work experiences shape retention/attrition decisions;<br>5) **Key predictors of retention:** job satisfaction, autonomy, leadership support, work–life balance, and professional development opportunities.		
**25**	**Pressley e Garside (2022)**	Safeguarding the retention of nurses: A systematic review on determinants of nurse's intentions to stay	Systematic review<br><br>**Aim:** to explore factors that influence registered nurses' intention to stay working in the healthcare sector.	34 studies were included. CINAHL, Medline and Cochrane library databases were searched from January 2010 to January 2022.	Two overarching factors influence intention to stay: job satisfaction and organizational commitment. 26 factors associated with these constructs were identified and grouped into three categories:<br>1) **Environmental factors** (e.g., work environment, organizational culture, safety climate, development opportunities);<br>2) **Relational factors** (e.g., leadership, teamwork, trust, job embeddedness, social support, belonging);<br>3) **Individual factors** (e.g., stress, burnout, emotional intelligence, resilience, autonomy, empowerment, professional values).<br><br>Leadership, empowerment, and supportive culture are critical to retention across all regions.	The heterogeneity of study designs prevented the conduct of a meta-analysis, and therefore the findings were synthesized narratively.<br><br>The inclusion of only English-language, peer-reviewed quantitative studies may have introduced publication bias and resulted in the exclusion of relevant grey literature.<br><br>The review's focus on nurses' intention to stay, rather than on actual retention behavior, limits the ability to draw causal inferences.<br><br>There was limited exploration of economic and policy-level interventions aimed at improving nurse retention.	**Quality Rating: High**
